# Vitamin A levels reflect disease severity and portal hypertension in patients with cirrhosis

**DOI:** 10.1007/s12072-020-10112-3

**Published:** 2020-12-08

**Authors:** Benedikt Simbrunner, Georg Semmler, Alexander Stadlmann, Bernhard Scheiner, Philipp Schwabl, Rafael Paternostro, Theresa Bucsics, David Bauer, Ernst Eigenbauer, Matthias Pinter, Albert-Friedrich Stättermayer, Peter Quehenberger, Rodrig Marculescu, Michael Trauner, Mattias Mandorfer, Thomas Reiberger

**Affiliations:** 1 Division of Gastroenterology and Hepatology, Department of Internal Medicine III, Medical University of Vienna, Währinger Gürtel 18-20, 1090 Vienna, Austria; 2 Vienna Hepatic Hemodynamic Laboratory, Medical University of Vienna, Vienna, Austria; 3 Ludwig Boltzmann Institute for Rare and Undiagnosed Diseases, Vienna, Austria; 4 CeMM Research Center for Molecular Medicine of the Austrian Academy of Sciences, Vienna, Austria; 5 Christian-Doppler Laboratory for Portal Hypertension and Liver Fibrosis, Medical University of Vienna, Vienna, Austria; 6 Klinikum Hietzing, Vienna, Austria; 7 IT4Science, Medical University of Vienna, Vienna, Austria; 8 Department of Laboratory Medicine, Medical University of Vienna, Vienna, Austria

**Keywords:** ACLD, Cirrhosis, Hepatic venous pressure gradient, Hepatic decompensation

## Abstract

**Background and Aims:**

The liver plays a key role in the storage, metabolism and homeostasis of fat-soluble vitamins. We investigated the relation of Vitamin(Vit)A/D/E serum levels with severity of liver disease and portal hypertension (PHT).

**Methods:**

VitA/D/E serum levels were assessed in 234 patients with advanced chronic liver disease (ACLD, i.e. hepatic venous pressure gradient [HVPG] ≥ 6 mmHg). Patients with hepatocellular carcinoma, pre-/post-hepatic PHT, TIPS or liver transplantation were excluded.

**Results:**

Most patients were male (*n* = 153; 65%) with a median age of 57.6 (49.7–64.5) years. Thirty-two (14%) patients had HVPG 6–9 mmHg, 66 (28%) 10-15 mmHg, and 136 (58%) ≥ 16 mmHg, respectively. VitD deficiency (25-OH-vitamin-D <50 nmol/L) was found in 133 (57%) with higher prevalence in Child-Turcotte-Pugh (CTP)-C: 85% vs. B: 66% vs. A: 47% (*p* < 0.001). VitD levels displayed significant but weak correlations with hepatic dysfunction and PHT. VitE levels were normal in 227 (97%) patients and displayed no relevant association with hepatic dysfunction or PHT. Only 63 (27%) patients had normal (>1.05 µmol/L) VitA levels, while 58 (25%) had mild (0.70–1.04 µmol/L), 71 (30%) moderate (0.35–0.69 µmol/L), and 42(18%) severe(<0.35 µmol/L) VitA deficiency. VitA correlated with HVPG (*Rho* = −0.409), CTP score (*Rho* = −0.646), and serum bile acid levels (Rho = −0.531; all *p* < 0.001). The prevalence of decompensated ACLD (dACLD) continuously increased with severity of VitA deficiency (no: 40% vs. mild: 51% vs. moderate: 67% vs. severe: 91% had dACLD; *p* < 0.001). CTP score (per point; OR 2.46; 95%CI 1.80–3.37; *p* <0.001), age (per year; OR 0.95; 95%CI 0.92–0.98; *p* = 0.001) and elevated bile acid levels(>10 µmol/L; OR 3.62; 95%CI 1.61–8.14; *p* = 0.002) were independently associated with VitA deficiency.

**Conclusion:**

VitA and VitD but not VitE deficiencies are highly prevalent in ACLD. VitA deficiency strongly correlates with hepatic dysfunction, PHT and bile acid levels and is associated with decompensated ACLD.

**Trial registration number:**

NCT03267615.

**Electronic supplementary material:**

The online version of this article (10.1007/s12072-020-10112-3) contains supplementary material, which is available to authorized users.

## Introduction

Fat-soluble vitamins A (retinol), D (cholecalciferol), E (tocopherols and tocotrienols), and K (phylloquinone and menaquinones) are lipophilic molecules with distinct physiological properties. While dietary intake is an important (or even exclusive) source for all vitamins, the lipophilicity of fat-soluble vitamins requires mediation of bile acids (BA) for intestinal uptake [1].

Dietary uptake is the exclusive source of vitamin A (VitA) in humans [2]. Notably, 60–95% of VitA is stored in the liver of healthy individuals, while only minor fractions are located in extrahepatic tissues [3]. Importantly, activated hepatic stellate cells (HSCs) are the main driver of fibrogenesis upon liver injury by producing extracellular matrix proteins. During their activation process, HSCs lose their lipid droplets containing retinyl esters [4, 5]. In patients with non-alcoholic fatty liver disease (NAFLD) undergoing bariatric surgery, reduced levels of serum and hepatic retinol, as well as retinoic acid (RA) have been observed, being inversely correlated with grade of hepatic steatosis and severity of non-alcoholic steatohepatitis (NASH) [6]. In other etiologies of (advanced) chronic liver disease, VitA deficiency was reported in a considerable percentage of patients and associated with higher fibrosis stages or cirrhosis [7–9].

Vitamin D (VitD) is mainly derived from the endogenous synthesis in the skin followed by two critical steps of hydroxylation in the liver and the kidneys [10]. Importantly, a previous study on patients with cirrhosis displayed a link between severe vitamin D deficiency (VitD_Def_) and increased levels of inflammatory biomarkers as well as the risk of hepatic decompensation [11]. Similarly, disease severity and mortality were associated with VitD_Def_ [12, 13].

Vitamin E (VitE) functions as an antioxidant by scavenging peroxyl radicals and regulating the oxidation process of polyunsaturated fatty acids, and is widely reported as a modulator and promoter of immunity [14]. However, while the direct effect of VitE on immunity-/oxidative stress-related pathways may be overestimated, prior studies have demonstrated an association between disease severity and oxidative stress in NAFLD patients [15].

This study aimed to investigate the prevalence of vitamin A, D, and E deficiencies in prospectively recruited patients with advanced chronic liver disease (ACLD) and to determine their association with severity of liver disease and portal hypertension (PHT). Furthermore, we assessed the previously unreported relation to serum levels of bile acids and fibrosis markers to address pathophysiological concepts of vitamin A homeostasis.

## Patients and methods

### Study design

234 patients with ACLD [defined by hepatic venous pressure gradient (HVPG) ≥ 6 mmHg] undergoing hepatic vein catheterization at the Vienna Hepatic Hemodynamic Lab of the Medical University of Vienna were consecutively included in the prospective VICIS study (NCT03267615) between 01/2017 and 03/2020. Patients with non-cirrhotic PHT, pre- or post-hepatic PHT, hepatocellular carcinoma, history of transjugular intrahepatic portosystemic shunt (TIPS) implantation or liver transplantation were excluded (Supplementary Fig. S1). Furthermore, patients under treatment with non-selective betablockers (NSBB) were excluded. More specifically, patients either had never received NSBB (including carvedilol) or paused NSBB intake 5 days before HVPG measurement. Patients’ medical records were reviewed to obtain relevant clinical information, laboratory parameters, and prescription vitamin supplements. Compensated ACLD (cACLD) was defined as the absence of hepatic decompensation events prior to HVPG measurement, i.e. ascites, hepatic encephalopathy, and variceal bleeding [16].

### Analysis of laboratory parameters

All reported laboratory parameters were assessed from blood samples obtained via the catheter introducer sheath placed in the internal jugular vein for HVPG measurement. Detailed information towards laboratory analyses is depicted in the supplementary material (“Supplementary Methods”).

### HVPG measurements and transient elastography

HVPG measurements were performed by trained physicians of the Vienna Hepatic Hemodynamic Lab following a defined standard operating procedure in fasting condition [17]. Detailed steps of the procedure are delineated in the supplementary material (“Supplementary Methods”).

### Statistics

Statistical analyses were performed using IBM SPSS Statistics 26 (IBM, Armonk, New York, USA) and GraphPad Prism 8 (GraphPad Software, La Jolla, California, USA). Continuous variables are reported as mean ± standard error of the mean (SEM) or median and interquartile range (IQR), and categorical variables are presented as numbers (*n*) and proportions (%) of patients. Comparisons of continuous variables were performed using Student’s *t* test or Mann–Whitney *U* test, as applicable. Post-hoc analysis was performed using Dunn’s multiple comparison test. Categorical variables were compared with Chi squared or Fisher‘s exact test, as applicable. Correlation between parameters were assessed by calculation of either Spearman or Pearson correlation coefficients dependent on the distribution of the respective parameters. Risk factors for moderate or severe vitamin A deficiency (VitA_Def_), or VitD_Def_, were calculated by uni- and multivariate logistic regression analysis, respectively. Parameters achieving a *p* value ≤ 0.10 in univariate analysis were subsequently included in multivariate analysis. In all analyses, a two-sided *p* value ≤ 0.05 was defined to denote statistical significance.

### Compliance with ethical standards

This study was conducted in accordance with the 1964 Helsinki declaration and its later amendments and approved by the local ethics committee of the Medical University of Vienna (EK1262/2017). All patients gave written informed consent to liver vein catheterizations and provided written consent to be enrolled in the VICIS study (NCT03267615). All authors had access to the study data and reviewed and approved the final manuscript.

## Results

### Patient characteristics

Most of the patients included in this study were male (*n* = 153/234, 65.4%), while the median age was 57.6 (49.7–64.5) years. Alcohol-related liver disease (ALD; *n* = 96, 41%) and viral hepatitis (*n* = 47, 20%) represented the predominant ACLD etiologies. Median HVPG was 18 (12–20) mmHg, and 202 (86%) patients had clinically significant portal hypertension (CSPH, i.e. an HVPG ≥ 10 mmHg). More specifically, HVPG was 6–9 mmHg in 32 patients (14%), 10–15 mmHg in 66 (28%) and ≥ 16 mmHg in 136 patients (58%). The majority of our study cohort was classified as Child-Turcotte-Pugh (CTP) stage A (*n* = 131, 56%), 83 (35%) CTP stage B, and 20 (9%) CTP stage C (Supplementary Table S1). Only 1 (0.4%) patient received prescription VitA supplements, whereas no prescription for VitE was recorded (of note, VitE is available as an “over-the-counter” supplement). Furthermore, 59 (25%) patients reported prescription VitD supplements. Twenty-one (9%) of these patients had VitD levels within the normal range, whereas 22 (9%) and 16 (7%) patients still had VitD insufficiency and deficiency, respectively. In only 6 (2.6%) patients, potential vitamin prescriptions were insufficiently recorded.

### Prevalence of vitamin A, D, and E deficiency

**Fig. 1:**
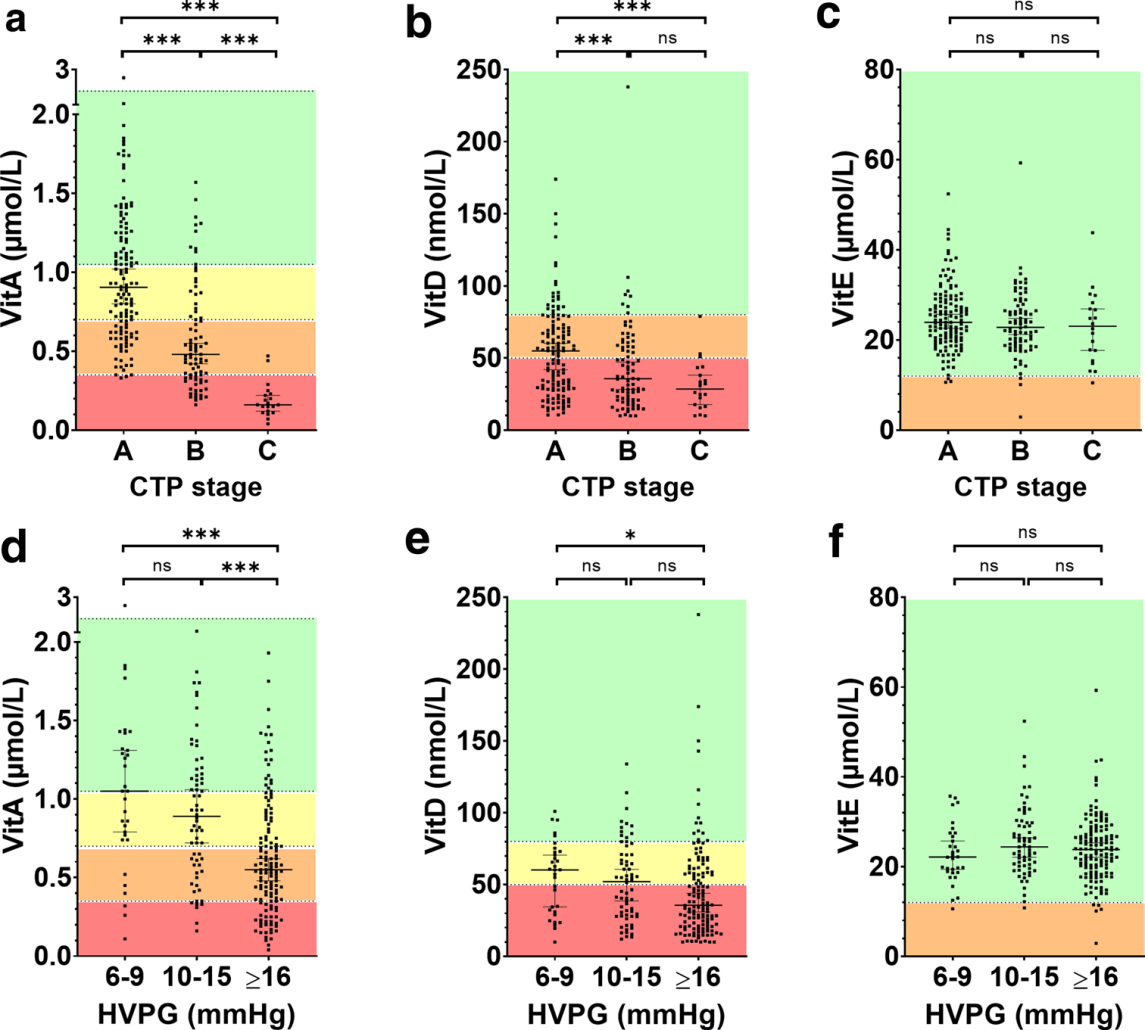
Vitamin A, D, and E serum levels in patients stratified by (**a**, ** b**, ** c**) Child-Turcotte-Pugh (CTP) stage and (**d**, ** e**,** f**) hepatic venous pressure gradient (HVPG). Different colours in the background indicate ranges between cut-offs for Vitamin A, D, and E deficiencies as specified in the methods section. *VitA* vitamin A, *VitD* vitamin D, *VitE* vitamin E, *HVPG* hepatic venous pressure gradient, *CTP* Child-Turcotte-Pugh, *ns* not significant, (*) *p* < 0.05, (***) *p* < 0.001

**Fig. 2:**
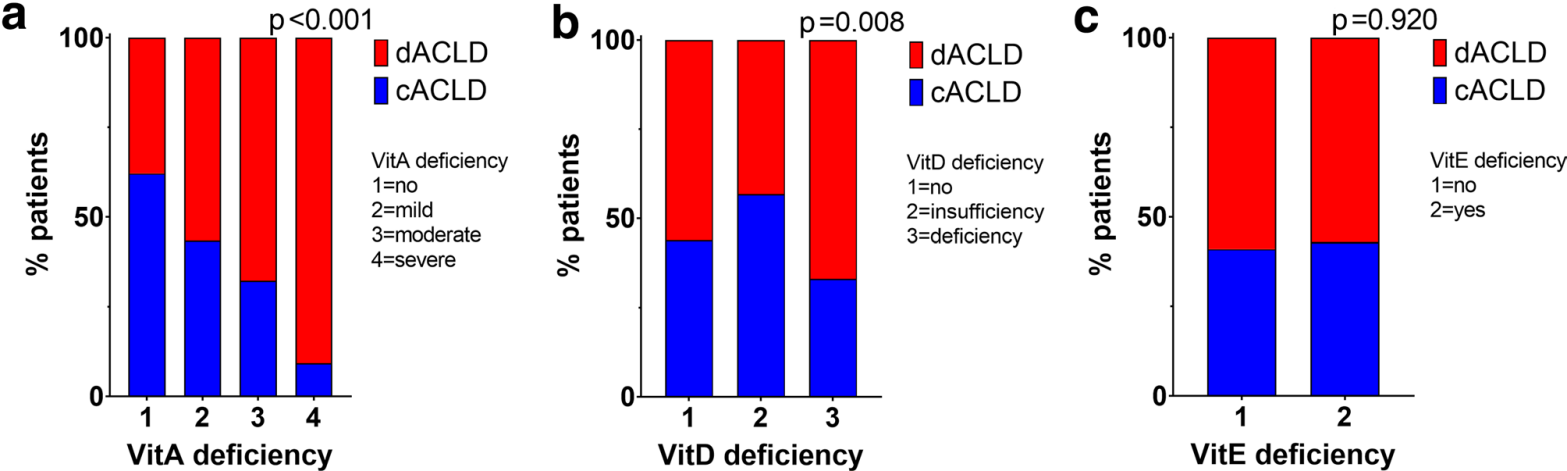
Prevalence of compensated and decompensated advanced chronic liver disease in patients stratified by the presence and severity of vitamin A, D, and E deficiency. *VitA* vitamin A, *VitD* vitamin D, *VitE* vitamin E, *cACLD* compensated advanced chronic liver disease, *dACLD* decompensated advanced chronic liver disease

The minority of patients (*n* = 63, 27%) in our study cohort had vitamin A serum levels within the normal range, whereas 53 (23%) had mild VitA_Def_, 76 (32%) had moderate VitA_Def_, and 42 (18%) had severe VitA_Def_. When stratifying patients by severity of PHT (i.e., 6–9, 10–15, ≥ 16 mmHg), VitA_Def_ prevalence incremented with rising HVPG (*p* < 0.001; Fig. 1). More importantly, stratification by CTP stage revealed a pronounced stepwise increase of VitA_Def_ severity across groups: 2 (1.5%) patients with CTP A, 22 (27%) with CTP B, and 18 (90%) with CTP C had severe VitA_Def_ (*p* < 0.001; Fig. 1a; Supplementary Table S2). Furthermore, the proportion of patients with decompensated ACLD (dACLD) continuously rose with VitA_Def_ severity grade as compared to compensated patients: 40% without VitA_Def_, 51% with mild, 67% with moderate, and 91% with severe VitA_Def_ had dACLD (*p* < 0.001; Fig. 2a).

Similarly, only 41 (17%) patients had VitD serum levels within the normal range, whereas 60 (26%) and 133 (57%) had VitD insufficiency and deficiency, respectively. VitD levels also gradually decreased across HVPG and CTP strata, however, the discrimination between groups was less pronounced as compared to VitA (Fig. 1e, *p* = 0.009; Fig. 2b, *p* = 0.008). Nevertheless, VitD_Def_ was observed among 62 (47%) patients with CTP A, 54 (66%) with CTP B, and 17 (85%) with CTP C (*p* < 0.001; Fig. 1b). Patients reporting VitD prescription supplementation had higher VitD levels across individual CTP and HVPG strata (Supplementary Fig. S2).

In contrast, vitamin E deficiency (VitE_Def_) was observed in only 7 (3%) patients. Severity of portal hypertension or disease stage was not associated with serum levels of VitE (Figs. 1c, f, 2c).

### Correlation between vitamin A, D, E levels and parameters of hepatic (dys-)function

**Fig. 3:**
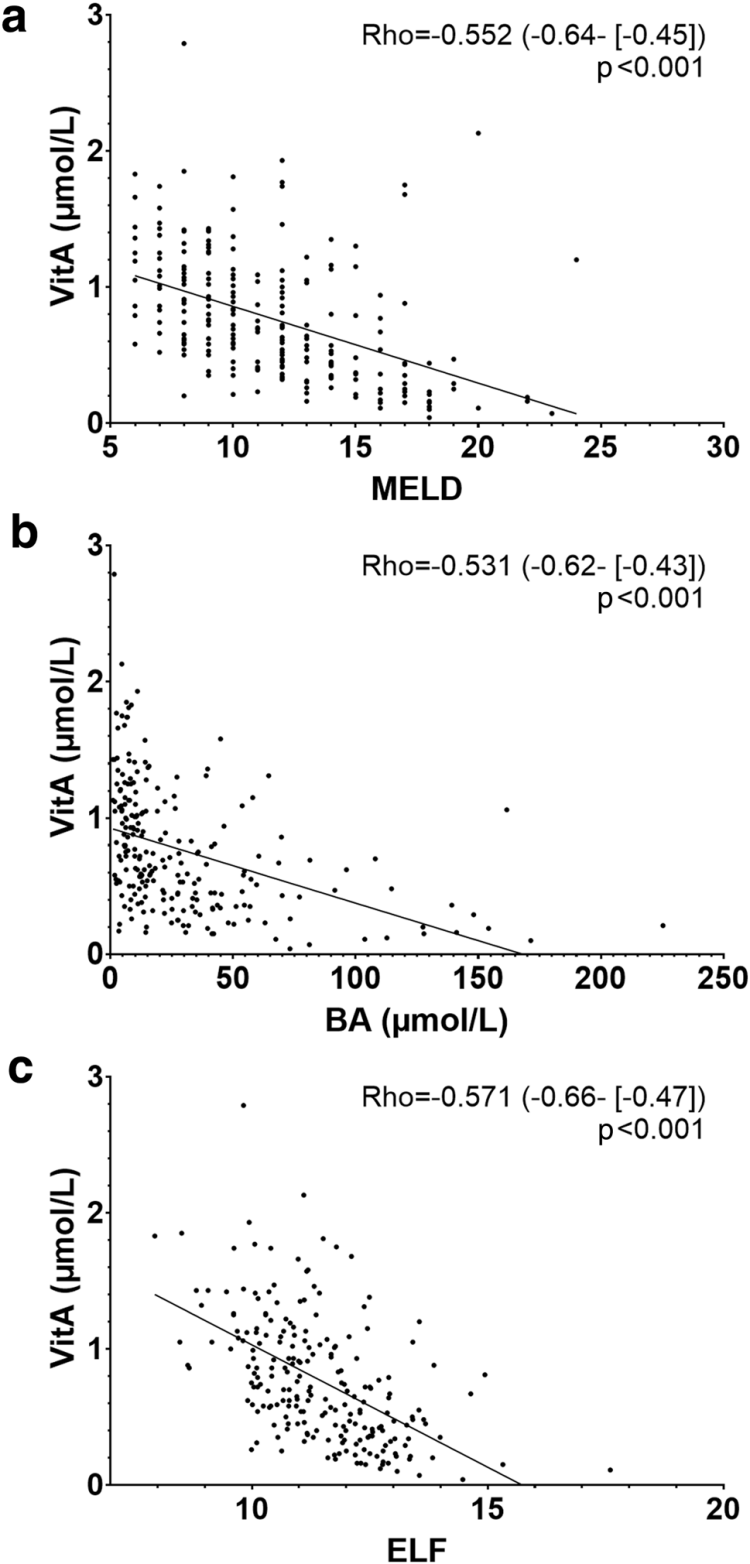
Correlation between vitamin A serum levels and Model for End-stage Liver Disease (MELD) score, bile acid serum levels, and enhanced liver fibrosis (ELF) score. *VitA* vitamin A, *MELD* Model for end-stage liver disease, *BA* bile acids, *ELF* enhanced liver fibrosis score

VitA serum levels showed a significant negative correlation with HVPG (Rho = −0.409, 95%CI: −0.51 to [−0.29]; *p* < 0.001), and even stronger association with MELD (Rho = −0.552, 95%CI: −0.64 to [−0.45]; *p* < 0.001; Fig. 3a) and CTP score (Rho = −0.646, 95%CI: −0.72 to [−0.56]; *p* < 0.001) (Table 1). Similarly, serum BA levels were significantly associated with VitA (Rho = −0.531, 95%CI −0.62 to [−0.43]; *p* < 0.001; Fig. 2b). Furthermore, surrogate parameters for liver fibrosis displayed inverse correlation with VitA: Rho = −0.393 (95%CI −0.52 to [−0.26]; *p* < 0.001; available in *n* = 174) for vibration-controlled transient elastography (VCTE) and Rho = -0.571 (95%CI −0.66 to [−0.47]; *p* < 0.001; Fig. 2c) for enhanced liver fibrosis score (ELF).

**Table 1:**
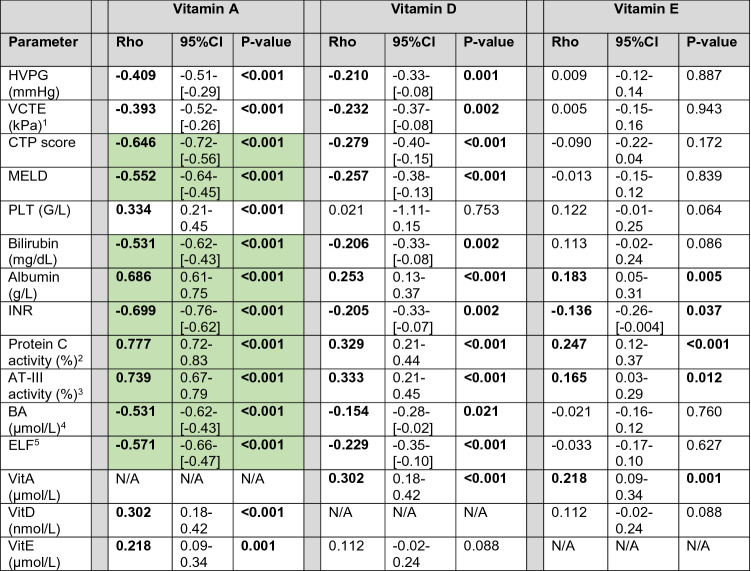
Correlation of Vitamin A, D, and E serum levels with hemodynamic and laboratory parameters

*p* values < 0.05 are indicated in bold. Correlation coefficients > 0.500/ < − 0.500 are indicated in bold and highlighted in green

*HVPG* hepatic venous pressure gradient, *CTP* Child-Turcotte-Pugh, *VCTE* vibration-controlled transient elastography, *MELD* Model for end-stage liver disease, *PLT* platelet count, *VitA* vitamin A, *VitD* vitamin D, *VitE* vitamin E, *INR* international normalized ratio, *AT-III* antithrombin III, *BA* bile acids, *ELF* enhanced liver fibrosis score

^1^Reliable VCTE results were available in *N* = 174 (74.4%) patients

^2^Protein C activity was available in *N* = 229 (97.9%) patients

^3^AT-III activity was available in *N* = 230 (98.3%) patients

^4^Bile acid serum levels were available in *N* = 224 (95.7%) patients

^5^ELF score was available in *N* = 219 (93.6%) patients

Furthermore, VitA levels strongly correlated with any single parameter of CTP and MELD scores reflecting hepatic synthesis (Table 1, Supplementary Fig. S3). As for the good correlation of VitA with Vitamin K-dependent coagulation parameters, i.e., INR (Rho = −0.699, 95%CI −0.76 to [−0.62], *p* < 0.001) and Protein C activity (Rho = 0.777, 95%CI 0.72–0.83, *p* < 0.001), we also observed strong association with vitamin K-independent antithrombin III (ATIII; Rho = 0.739, 95%CI 0.67–0.79; *p* < 0.001) (Supplementary Fig. S3).

VitD levels displayed significant but weak correlations with PHT and hepatic dysfunction. Similarly, weak but statistically significant associations were found for parameters reflecting hepatic synthesis capacity (Table 1).

Lastly, we observed no meaningful associations between VitE and PHT, disease severity, or hepatic synthesis. Furthermore, neither BA levels nor ELF score showed a significant correlation with VitE (Table 1).

### Differences in hepatic (dys-)function between high and low vitamin A quintiles

Due to the strong association between hepatic dysfunction/disease severity and VitA, we aimed to assess differences between patients stratified by high and low quintiles of VitA serum levels (Supplementary Table S3). Consequently, quintile 1 (Q1) represented patients with the lowest 20% of VitA levels, Q2-Q4 represented percentiles 20–80, and Q5 represented patients with the highest 20% VitA levels.

Interestingly, both age and sex displayed significant differences between low and high quintiles. Patients in Q5 (i.e. high VitA levels) were older than patients in lower quintiles (63.7 vs. 54.0 years; *p* = 0.001). In contrast, male patients were overrepresented in Q5, indicating that women had significantly lower levels of vitamin A (male sex 79.6% vs. 55.1%; *p* = 0.034).

Concordantly, HVPG (median 19 mmHg in Q1 vs. 12 mmHg in Q5), CTP (8 points in Q1 vs. 5 points in Q5) and MELD (16 points in Q1 vs. 9 points in Q5) score, as well as the prevalence of dACLD (84% in Q1 vs. 41% in Q5) significantly differed between quintiles (all *p* < 0.001).

**Fig. 4:**
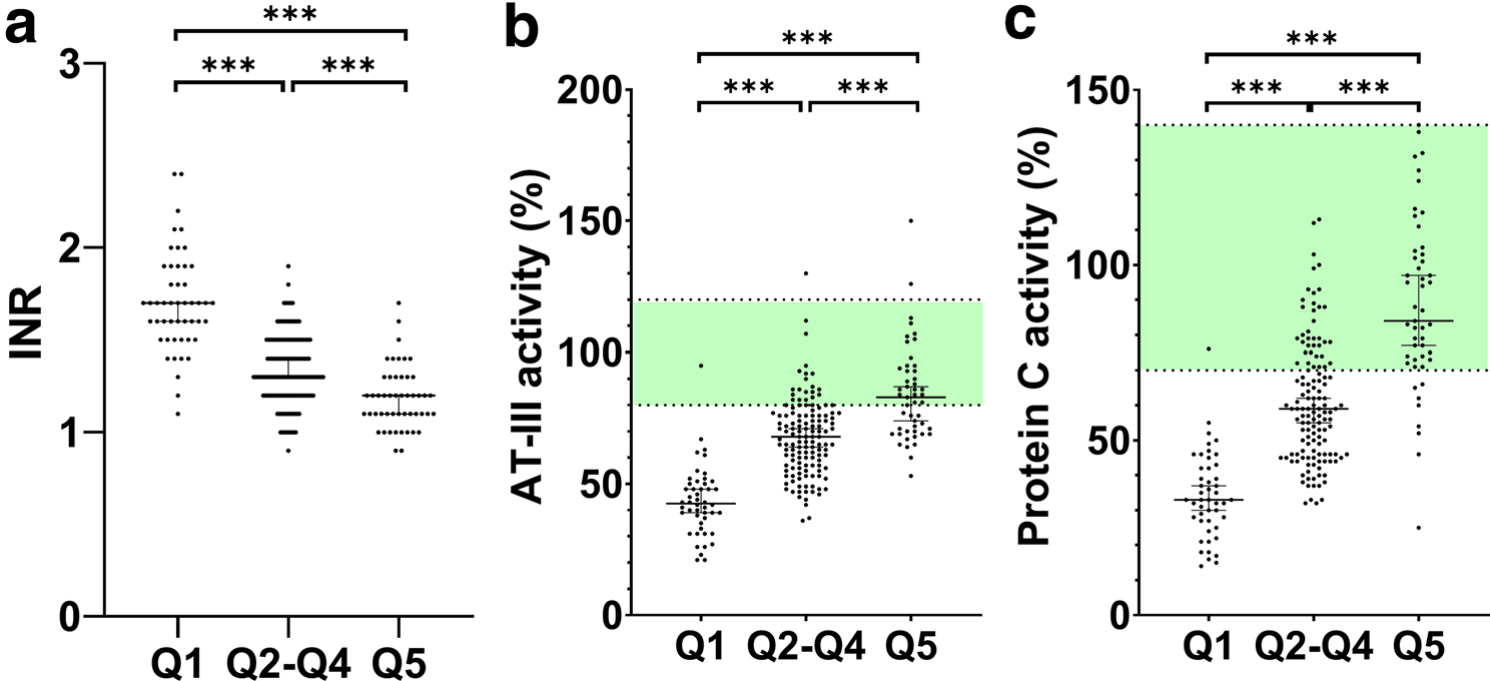
Comparison of coagulation parameters in patients stratified by vitamin A quintiles. Green colour in the background indicates normal ranges for AT-III and Protein C activity, respectively. *Q1–Q5* quintile 1–5; *INR* international normalized ratio; *AT-III* antithrombin-III, (***) *p* < 0.001

Similarly, VitK-dependent and -independent coagulation parameters displayed strong differences between VitA quintiles (Fig. 4): median INR yielded 1.7 in Q1 vs. 1.2 in Q5, AT-III activity was almost half with 43% in Q1 vs. 83% in Q5, similar to protein C activity with 33% in Q1 and 84% in Q5 (all *p* < 0.001).

Conversely, body-mass index (BMI) as a basic parameter for cachexia/obesity/nutritional status was similar between vitamin A strata (*p* = 0.121).

### Independent risk factors for vitamin A, D, and E deficiency

Furthermore, independent risk factors for vitamin A/D/E deficiencies were assessed using binary logistic regression analysis (Table 2). Age (per year; OR 0.97, 95% CI 0.94–0.99, *p* = 0.003), HVPG (per mmHg; OR 1.16, 95%CI 1.10–1.22, *p* < 0.001), CTP score (per point; OR 2.44, 95% CI 1.89–3.15, *p* < 0.001), and elevated serum BA (OR 7.06, 95% CI 3.73–13.4, *p* < 0.001) were associated with VitA_Def_ on univariate analysis (Table 2). On multivariate analysis, CTP score (per point; OR 2.25, 95% CI 1.67–3.04, *p* < 0.001) and elevated serum BA levels (OR 3.53, 95% CI 1.61–7.76, *p* = 0.002) emerged as independent risk factors for VitA_Def_, whereas age (per year; OR 0.95, 95% CI 0.92–0.98, *p* = 0.001) was associated with the absence of moderate or severe VitA_Def_.

**Table 2:**
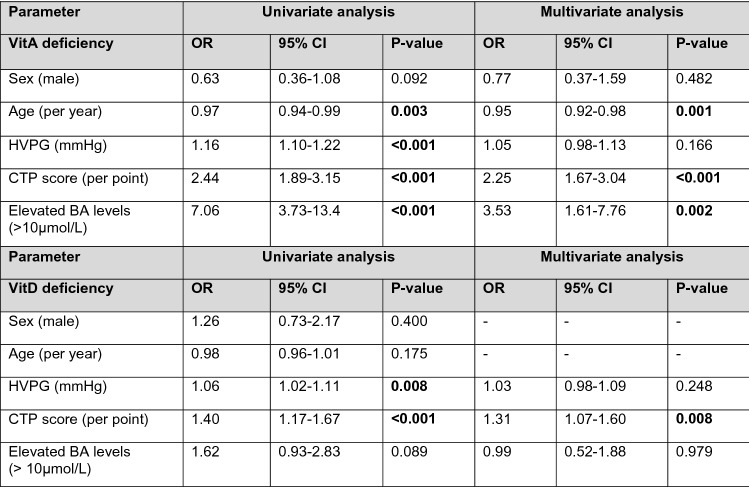
Independent risk factors for moderate or severe vitamin A deficiency and vitamin D deficiency

*p* values < 0.05 are indicated in bold

*OR* odds ratio, *95%CI* 95% confidence interval, *HVPG* hepatic venous pressure gradient, *CTP* Child-Turcotte-Pugh, *BA* bile acids

Similarly, HVPG (per mmHg; OR 1.06, 95%CI 1.02–1.11, *p* = 0.008) and CTP score (per point; OR 1.40, 95% CI 1.17–1.67, *p* < 0.001) but not age or serum BA levels–were significantly associated with VitD_Def_ on univariate analysis (Table 2). Only CTP score remained an independent risk factor for VitD_Def_ (per point; OR 1.31, 95% CI 1.07–1.60, *p* = 0.008).

Importantly, when performing these analyses using either HVPG or CTP score only, both of these parameters emerged as independent risk factors for VitA and VitD deficiency, respectively, which might be attributed to collinearity between HVPG and CTP score in reflecting the severity of liver disease (Supplementary Tables S4, S5).

Conversely, we were not able to identify any risk factor for VitE deficiency (Supplementary Table S6).

### Independent risk factors for decompensated liver disease

Since VitA and VitD deficiency was more prevalent in patients with dACLD, we assessed whether VitA and VitD levels were independently associated with dACLD (Supplementary Table S7). We chose to use MELD for this analysis of dACLD patients only, as the decompensation events ascites and encephalopathy are included in the CTP score. On univariate analysis, HVPG and MELD, as well as VitA and VitD serum levels emerged as risk factors for dACLD. However, only MELD (per point; OR 1.22, 95%CI 1.09–1.36; *p* < 0.001) and HVPG (per mmHg; OR 1.19, 95%CI 1.11–1.27; *p* < 0.001) remained independently associated with dACLD.

## Discussion

This study is the first to simultaneously describe VitA/D/E status in a large prospective cohort of 234 ACLD patients with characterized severity of PHT. We found a VitA_Def_ in 73% of our patients and its prevalence increased with CTP stages and was associated with dACLD. Similarly, 57% had VitD_Def_, which also displayed a distinct association with disease severity. Conversely, we found that only 3% had low VitE serum levels and did not observe an association of VitE levels with liver dysfunction or PHT severity. Of note, our study provides novel data by investigating the relationship of these fat-soluble vitamins with serum BA levels as well as ELF score (both available in about 95% of patients), which is a well-established and an increasingly used non-invasive surrogate parameter for fibrosis.

The liver represents the key regulator of VitA homeostasis, its storage in HSCs, intestinal absorption by BA production and its systemic distribution by the synthesis of retinol-binding protein 4 (RBP4) and transthyretin [18]. As mentioned above, prior studies on patients with NAFLD reported reduced VitA levels both in the serum and the liver [6], being inversely correlated with the grade of hepatic steatosis and severity of NASH [19]. It is reasonable to hypothesize that during the progression of fibrosis, HSCs lose their essential ability to store VitA, subsequently leading to progressive VitA_Def_ as fibrosis accumulates in the liver. Ultimately, this process might be reflected by reduced VitA serum levels. This assumption is supported by data from the literature [7–9], as well as the moderate/strong correlation of VitA levels with markers of hepatic dysfunction and, importantly, ELF score, in our study. However, it has to be acknowledged that VitA serum levels may not mirror hepatic VitA content [20].

Furthermore, VitA_Def_ may also be due to inadequate intake or intestinal malabsorption. In this regard, it seems very interesting that elevated bile acids were independently associated with VitA_Def_. It might be argued that serum levels might not reflect the availability of bile acids in the intestines and that impaired bile acid homeostasis and biliary excretion may rather reflect impaired liver function in cirrhosis [21]. Intriguingly, retinoic acid is required for activation of retinoid X receptor-alpha (RXR-α) as a heterodimeric partner of the farnesoid X receptor (FXR) and other key nuclear receptors required for transactivation of genes maintaining proper bile acid homeostasis and excretion [22]. Importantly, elevated serum BA levels may also be a reflection of portosystemic shunting, and thereby, the degree of PHT [23, 24].

Dietary intake of VitA was not systematically assessed in our patients, which represents an important limitation of this study. Interestingly, a previous study on patients with cirrhosis reported that adequate VitA intake did not prevent VitA deficiency [25]. Similarly, another study on patients with (A)CLD applied the relative-dose-response method to assess hepatic VitA storages after oral VitA challenge and reported that neither duration nor dosage of VitA supplementation was independently associated with an effective test response. In contrast, low RBP was the only independent parameter associated with failed test response [26]. Since RBP is synthesized in the liver, it might be hypothesized that VitA serum levels are closely associated with its synthetic capacity and that low serum VitA levels are simply a consequence of impaired hepatic function. Several findings of our study support this hypothesis: First, CTP score – reflecting important aspects of hepatic dysfunction was independently associated with VitA_Def_. Second, we observed a moderate/strong correlation of VitA with all laboratory parameters reflecting hepatic synthesis capacity, including vitamin K-dependent and independent coagulation parameters. For example, AT-III and Protein C activity were reduced by nearly 50% in the lowest vs. highest VitA quintile. Third, the independent association of CTP score with VitD (25-OH-vitamin D) deficiency further underlines the role of hepatic dysfunction towards decreased vitamin serum levels as this metabolic step in the course of VitD synthesis is localized in the liver [10].

Finally, we observed that patients with low VitA levels were significantly younger than patients with high VitA levels, which was also confirmed on multivariate regression analysis. At this point, we can only speculate that patients undergoing HVPG measurement (i.e., had developed ACLD) at a younger age had a more progressive course of the disease, which might be related to pronounced VitA depletion.

Correction of VitA levels in ACLD, however, is challenging: First, simple supplementation might not lead to effective amelioration of VitA serum levels, depending on the severity of liver disease [25, 26]. Second, it remains unclear whether VitA_Def_ (as assessed by serum VitA levels) really impacts on downstream signaling relevant for modulation of liver disease. Lastly, overdosing of VitA may cause hepatotoxicity which may be of particular concern in patients with ACLD [27]. However, the strong link between VitA_Def_ and fibrosis may indicate that treatment strategies promoting fibrosis regression will also ameliorate VitA homeostasis and vice versa.

VitD_Def_ was highly common in our study cohort, being present in 57% of patients and additional 26% with VitD insufficiency, according to commonly used cut-offs for 25-OH-vitamin D levels [10]. Of note, comparisons to other studies regarding the prevalence of VitD_Def_ are difficult to draw, as there are considerable differences in cut-offs and patient characteristics, however, other studies have demonstrated similar results [11, 12]. As indicated previously, a recent meta-analysis confirmed the association between VitD deficiency and mortality in patients with cirrhosis [13].

In our study, VitD levels were clearly associated with disease stage and severity of PHT. As mentioned above, only severity of hepatic dysfunction (i.e. CTP score) remained an independent risk factor for VitD_Def_, which seems particularly interesting in regard to the observation that elevated BA levels were independently associated with VitA_Def_ but not with VitD_Def_. While VitA supply is exclusively dependent on dietary uptake (mediated by BA [1]), VitD is mainly provided by endogenous synthesis/metabolization [10]. Therefore, the identification of hepatic (dys-)function – but not elevated BA levels – as the main determinant for 25-OH-vitamin D deficiency seems consistent with physiological concepts of VitD metabolism.

Importantly, a previous RCT by Pilz et al. demonstrated that VitD supplementation effectively increases VitD serum levels in patients with cirrhosis [28], while another study reported rapid declines of VitD levels after cessation of supplementation [29]. Furthermore, the efficacy of VitD supplementation towards osteopenia/osteoporosis remains unclear, as parathyroid hormone levels were not affected by VitD supplementation in the study by Pilz et al [28] and since several studies displayed no benefits of VitD supplementation on bone density/osteoporosis in patients with liver disease [30–32]. Similarly, a systematic review on VitD supplementation in chronic liver disease suggested limited efficacy of VitD supplementation on mortality, but importantly no effects on morbidity and health-related quality of life [33]. Nevertheless, in the absence of significant toxicity related to VitD supplementation in patients with ACLD, VitD supplementation seems safe, and thus, it may be argued that ACLD patients with VitD deficiency should still receive VitD supplements in case of low VitD levels.

Interestingly, our study clearly refutes that VitE_Def_ is a regular or relevant condition in ACLD. First of all, only 7 (3%) patients fulfilled laboratory criteria for VitE_Def_. This observation is particularly relevant in regard to previous studies reporting lower levels of VitE in patients with NASH as compared to healthy subjects [34]. Of note, the study by Erhardt et al. [34] reported mean VitE levels of 22.4 µmol/L and 26.8 µmol/L in NASH patients and controls, respectively, which was quite similar to our study (median 23.6 µmol/L). Therefore, we can only speculate whether “significantly” lower VitE serum levels – however, still within normal range between healthy controls and patients have an impact on disease or may even be classified as VitE_Def_ based on their potential implications on ACLD. Nevertheless, RCT displayed a significantly higher rate of histological improvement upon VitE treatment as compared to placebo in NASH patients [35], which was also suggested by a meta-analysis on VitE supplementation in NASH [36]. Similarly, a recent publication reported a remarkable reduction of risk for death or liver transplantation in compensated NASH patients receiving VitE supplements (adjusted HR 0.30) [37]. However, next to the retrospective and non-randomized study design, adherence to VitE intake in the treatment group was not assessed [37]. In any case, we did not observe any differences in VitE levels across CTP or HVPG strata or any meaningful correlation with parameters of hepatic (dys-)function. Of note, our study only included 25 (11%) NASH patients that exclusively displayed VitE levels above the cut-off for VitE_Def_.

Our study has several limitations: First, we could not provide data on hepatic VitA content, as well as a profile of different VitA metabolites in serum. Second, specific binding proteins for these vitamins were not measured, which prevents us from reporting their relation to vitamin serum levels. Third, we did not perform a systematic assessment of dietary vitamin intake, which would include both regular food intake as well as over-the-counter vitamin supplements, as well as current adherence towards and prior duration of prescription vitamin supplement intake. Fourth, the limited duration of potential follow-up in our study cohort prevents us from reporting follow-up events such as mortality and (further) hepatic decompensation in these patients at this point, which would have allowed to assess the prognostic value of VitA/D/E deficiencies. While these limitations must be acknowledged, we provide novel and important data on the strong association between vitamin deficiencies and ACLD severity as well as the severity of PHT in a large series of prospectively recruited patients. Furthermore, this study displays a close link between VitA levels and ELF score, as well as an independent association between elevated BA levels and VitA_Def_.

In summary, our study demonstrates a considerable prevalence of VitA and VitD deficiency in patients with ACLD, while VitE deficiency was uncommon. VitA and VitD deficiencies are closely linked to hepatic dysfunction, which may be explained by advanced fibrosis and/or synthetic dysfunction impacting on the uptake, storage, and metabolism of these vitamins. The prognostic value of VitA as well as its role in liver disease progression should be assessed in future studies.

## Electronic supplementary material

Below is the link to the electronic supplementary material.

Supplementary file1 (DOCX 1366 KB)

## Abbreviations

ACLD: Advanced chronic liver disease
ATIII: Antithrombin III
BA: Bile acids
BMI: Body-mass index
cACLD/dACLD: Compensated/decompensated advanced chronic liver disease
CSPH: Clinically significant portal hypertension
CTP: Child-Turcotte-Pugh
ELF: Enhanced liver fibrosis score
FXR: Farnesoid X receptor
HSC: Hepatic stellate cells
HVPG: Hepatic venous pressure gradient
IQR: Interquartile range
MELD: Model for end-stage liver disease
NAFLD: Non-alcoholic fatty liver disease
NASH: Non-alcoholic steatohepatitis
NSBB: Non-selective betablockers
PHT: Portal hypertension
RA: Retinoic acid
RXR-α: Retinoid X receptor-alpha
SEM: Standard error of the mean
TIPS: Transjugular intrahepatic portosystemic shunt
VCTE: Vibration-controlled transient elastography
VitA: Vitamin A
VitA_Def_: Vitamin A deficiency
VitD: Vitamin D
VitD_Def_: Vitamin D deficiency
VitE: Vitamin E
VitE_Def_: Vitamin E deficiency
