# Supplementary material for: Vitamin A levels reflect disease severity and portal hypertension in patients with cirrhosis
Source: Hepatol Int. 2020 Dec 8;14(6):1093–103. doi: 10.1007/s12072-020-10112-3 (PMC7803875; doi:10.1007/s12072-020-10112-3)
Supplement: Supplementary file 1 — Supplementary file1 (DOCX 1366 KB) [file 12072_2020_10112_MOESM1_ESM.docx]

**SUPPLEMENTARY MATERIAL**

**SUPPLEMENTARY METHODS**

*HVPG measurements and transient elastography*

HVPG measurements were performed by trained physicians of the Vienna Hepatic Hemodynamic Lab following a defined standard operating procedure in fasting condition [1]. Briefly, the right internal jugular vein was punctured by ultrasound guidance under local anaesthesia. A catheter introducer sheath (8.5 F, Arrow International, Reading, PA, USA) was inserted using Seldinger technique. The liver vein was cannulated by an angled balloon occlusion catheter (Medical University of Vienna/Medizintechnik Pejcl, Austria). Adequate placement and wedge position were verified by X-ray after injection of contrast agent while the balloon was inflated. At least three measurements of free and wedged hepatic vein pressure were performed to assess HVPG.

Vibration-controlled transient elastography (VCTE; Echosense, Paris, France) was performed prior to HVPG measurements, applying reliability criteria as previously described [2].

*Analysis of vitamin A, D, and E serum levels*

Vitamin analyses were performed at the Department of Laboratory Medicine, Medical University of Vienna according to ISO 9001 and ISO 15189 quality standards. Serum VitA (Retinol) and VitE were measured by HPLC with UV detection using in vitro diagnostics certified (IVD CE) kit from ChromSystems (ChromSystems Instruments & Chemicals GmbH, Gräfeling, Germany) according to the manufacturer’s instructions. HPLC was performed on a Prominence Modular HPLC system (Shimadzu, Kyoto, Japan). The laboratory’s reference ranges of 1.05 - 2.45 µmol/L for VitA and 12 – 42 µmol/L for VitE were adopted. The intra-assay coefficients of variation for VitA were 2.5%, 0.8% and 0.9% at 0.47 mg/l, 0.66 mg/l and 1.27 mg/l, respectively. The inter-assay coefficients of variation for VitA were 3.8% and 5.0% at 0.40 mg/l and 0.98 mg/l, respectively. VitA deficiency (VitA_Def_) was further subclassified as mild (serum levels between 0.70 and 1.04 µmol/L), moderate (between 0.35 and 0.69 µmol/L), and severe (below 0.35 µmol/L) [3]. Serum 25-hydroxy-vitamin D was measured by the Liaison Total 25-Hydroxy-Vitamin D chemiluminescence immunoassay (CLIA) on Liaison XL immunology analyzers (DiaSorin, Saluggia, Italy). Vitamin D insufficiency was defined as serum levels between 50 and 80 nmol/L, and VitD_Def_ as serum levels below 50 nmol/L [4].

*Analysis of enhanced liver fibrosis score and bile acid serum levels*

Serum measurements of hyaluronic acid, tissue inhibitor of metalloproteinase 1 (TIMP-1) and type III pro-collagen N-terminal pro-peptide (PIIINP) for the enhanced liver fibrosis (ELF) test were performed by the corresponding IVD CE CLIAs on Advia Centaur CP analyzers (Siemens Healthcare GmbH, Erlangen, Germany). Measurement of enhanced liver fibrosis (ELF) score was available in 219 (94%) patients. Of note, ELF data from a subset of this study cohort have been reported previously [5]. Bile acids (BA) were measured in serum by direct spectrophotometry in the ISO-certified laboratory of the Medical University of Vienna according to the manufacturer’s instructions. BA levels above 10 µmol/L were considered beyond the upper limit of normal (available in N=224, 96%). Measurements were performed by technicians at the department of Laboratory Medicine at the Medical University of Vienna without access to clinical and hemodynamic parameters of included patients.

**SUPPLEMENTARY TABLES**

**Supplementary Table-S1. Patient characteristics.**

| **Parameter** | **N=234** |
| --- | --- |
| Age (years) | 57.6 (49.7-64.5) |
| Sex (M, %) | 153 (65.4) |
| Etiology (n, %)   - ALD - Viral  SVR  Viremic - Other  ALD + Viral  SVR  Viremic  NASH  Cholestatic (PBC/PSC)  AIH  Mixed etiologies^1^  Cryptogenic  Wilson’s disease  HFE | 96 (41.0)  47 (20.1)  30 (63.8)  17 (36.2)  91 (38.9)  18 (7.7)  12 (66.7)  6 (33.3)  25 (10.7)  9 (3.8)  8 (3.4)  11 (4.7)  18 (7.7)  1 (0.4)  1 (0.4) |
| HVPG (mmHg)   - 6-9 (n, %) - 10-15 (n, %) - ≥ 16 (n, %) | 18 (12-20)  32 (13.7)  66 (28.2)  136 (58.1) |
| CSPH (n, %) | 202 (86.3) |
| cACLD (n, %) | 93 (39.7) |
| Varices (n, %)   - None - Small - Large - Unknown | 79 (33.8)  60 (25.6)  88 (37.6)  7 (3.0) |
| History of variceal bleeding (n, %) | 23 (9.8) |
| History of endoscopic band ligation (n, %) | 42 (17.9) |
| Ascites (n, %)   - None - Mild/medically controlled - Severe/refractory | 128 (54.7)  91 (38.9)  15 (6.4) |
| Hepatic encephalopathy (n, %)   - None - Mild/medically controlled - Severe | 187 (79.9)  47 (20.1)  0 (0) |
| CTP Score (points) | 6 (5-8) |
| CTP Stage   - A - B - C | 131 (56.4)  83 (35.0)  20 (8.5) |
| MELD Score (points) | 11 (9-14) |
| VCTE (kPa)^2^ | 32.4 (18.3-61.6) |
| Vitamin A (µmol/L)  - No deficiency (n, %)  - Mild deficiency (n, %)  - Moderate deficiency (n, %)  - Severe deficiency (n, %) | 0.69 (0.42-1.06)  63 (26.9)  53 (22.6)  76 (32.5)  42 (17.9) |
| Vitamin D (nmol/L)   - No deficiency (n, %) - Insufficiency (n, %) - Deficiency (n, %) | 42.6 (24.8-66.4)  41 (17.5)  60 (25.7)  133 (56.8) |
| Vitamin E (µmol/L)   - No deficiency (n, %) - Deficiency (n, %) | 23.6 (19.6-28.3)  227 (97.0)  7 (3.0) |
| BMI (kg/m^2^) | 26.7 (23.5-30.9) |
| PLT (G/L) | 101 (71-141) |
| Creatinine (mg/dL) | 0.73 (0.60-0.94) |
| Albumin (g/L) | 36.9 (33.2-40.2) |
| INR | 1.3 (1.2-1.5) |
| AT-III activity (%)^3^ | 66 (52-78) |
| Protein-C activity (%)^4^ | 58 (44-77) |
| BA (µmol/L)^5^ | 14.9 (7.5-39.1) |
| ELF^6^ | 11.3 (10.5-12.4) |

^1^ Combinations of different etiologies associated with chronic liver disease such as NASH, viral hepatitis, A1ATD, and ALD. ^2^ Reliable VCTE results were available in N=174 (74.4%) patients.
^3^ AT-III activity was available in N=230 (98.3%) patients.
^4^ Protein C activity was available in N=229 (97.9%) patients.
^5^ Bile acid serum levels were available in N=224 (95.7%) patients.
^6^ ELF score was available in N=219 (93.6%) patients.

Abbreviations: (M) Male sex; (ALD) Alcohol-related liver disease; (NASH) Non-alcoholic steatohepatitis; (PBC) Primary biliary cholangitis; (PSC) Primary sclerosing cholangitis; (AIH) Autoimmune hepatitis; (A1ATD) Alpha-1 antitrypsin deficiency; (HFE) Hemochromatosis; (dACLD) decompensated advanced chronic liver disease; (CTP) Child-Turcotte-Pugh; (MELD) Model for end-stage liver disease; (HVPG) Hepatic venous pressure gradient; (TE) transient elastography; (BMI) body-mass index; (INR) International normalized ratio; (PLT) Platelet count; (vWF) von Willebrand factor; (TPZ) thromboplastin time; (AT-III) antithrombin-III; (BA) bile acid; (ELF) Enhanced liver fibrosis score

**Supplementary table-S2. Patient characteristics stratified by Child-Turcotte-Pugh (CTP) stage.**

| **N=234** | **CTP-A (N=131)** | **CTP-B (N=83)** | **CTP-C (N=20)** | **P-value** |
| --- | --- | --- | --- | --- |
| Vitamin A (µmol/L) | 0.91 (0.62-1.24) | 0.48 (0.33-0.76) | 0.16 (0.11-0.23) | **<0.001** |
| Vitamin D (nmol/L) | 54.9 (29.2-71.0) | 35.6 (21.1-59.3) | 28.5 (16.7-42.0) | **<0.001** |
| Vitamin E (µmol/L) | 23.9 (20.0-28.9) | 22.8 (18.8-26.9) | 23.1 (16.0-28.0) | 0.327 |
| MELD (points) | 9 (8-11) | 13 (12-15) | 18 (17-19) | **<0.001** |
| HVPG (mmHg) | 14 (10-19) | 19 (16-21) | 22 (18-26) | **<0.001** |
| Age (years) | 57.9 (50.2-65.0) | 57.5 (49.4-65.2) | 53.6 (49.7-61.8) | 0.632 |
| Sex (M, %) | 85 | 58 | 10 (50) | 0.241 |
| VCTE (kPa)^1^ | 22.3 (15.3-35.8) | 50.0 (29.2-75.0) | 73 (40.3-75) | **<0.001** |
| Etiology (n, %)   - ALD - Viral - Mix (ALD + viral) - NASH - CHOL - OTHER | 38 (29)  36 (27)  9 (7)  20 (15)  7 (5)  21 (16) | 46 (55)  6 (7)  8 (10)  4 (5)  2 (2)  17 (20) | 12 (60)  5 (25)  1 (5)  1 (5)  0 (0)  1 (5) | **<0.001** |
| Varices (n, %)   - None - Small - Large - (Unknown) | 51 (39)  33 (25)  40 (31)  7 (5) | 22 (27)  21 (25)  40 (48)  0 (0) | 6 (30)  6 (30)  8 (40)  0 (0) | 0.158 |
| BMI (kg/m^2^) | 27.1 (23.7-31.2) | 26.3 (22.6-29.9) | 26.1 (23.6-31.7) | 0.361 |
| PLT (G/L) | 105 (72-144) | 97 (71-135) | 91 (57-129) | 0.466 |
| Albumin (g/L) | 39.3 (37.0-41.1) | 33.8 (31.0-36.1) | 26.6 (25.1-30.2) | **<0.001** |
| Bilirubin (mg/dL) | 0.84 (0.61-1.19) | 1.4 (0.95-2.14) | 3.22 (2.42-4.05) | **<0.001** |
| INR | 1.2 (1.1-1.4) | 1.5 (1.3-1.6) | 1.8 (1.7-2.1) | **<0.001** |
| AT-III activity (%)^2^ | 74 (65-85) | 56 (46-69) | 40 (31-48) | **<0.001** |
| Protein-C activity (%)^3^ | 71 (56-88) | 46 (39-60) | 28 (18-33) | **<0.001** |
| BA (µmol/L)^4^ | 10.0 (5.9-22.1) | 27.4 (12.5-49.3) | 70.3 (27.4-127.7) | **<0.001** |
| ELF^5^ | 10.8 (10.1-11.5) | 12.0 (11.1-12.6) | 13.0 (12.8-13.6) | **<0.001** |

^1^ Reliable VCTE results were available in N=174 (74.4%) patients.
^2^ AT-III activity was available in N=230 (98.3%) patients.
^3^ Protein C activity was available in N=229 (97.9%) patients.
^4^ Bile acid serum levels were available in N=224 (95.7%) patients.
^5^ ELF score was available in N=219 (93.6%) patients.

P-values <0.05 are indicated in bold. Abbreviations: (M) male sex, (ALD) alcohol-related liver disease, (NASH) non-alcoholic steatohepatitis, (CHOL) cholestatic liver disease, (HVPG) hepatic venous pressure gradient, (VCTE) vibration-controlled transient elastography, (BMI) body-mass index, (MELD) Model for end-stage liver disease, (PLT) platelet count, (INR) international normalized ratio, (TPZ) thromboplastin time, (vWF) von Willebrand factor, (AT-III) antithrombin III, (BA) bile acids, (ELF) enhanced liver fibrosis score

**Supplementary table-S3. Patient characteristics stratified by high and low quintiles of vitamin A serum levels.**

| **N=234** | **Q1 (N=49)** | **Q2-Q4 (N=136)** | **Q5 (N=49)** | **P-value** |
| --- | --- | --- | --- | --- |
| Age (years) | 54.0 (45.6-61.1) | 57.4 (49.5-62.8) | 63.7 (52.9-69.2) | **0.001** |
| Sex (M, %) | 27 (55.1) | 87 (64.0) | 39 (79.6) | **0.034** |
| BMI (kg/m^2^) | 26.4 (22.6-31.4) | 26.4 (22.8-30.6) | 28.3 (24.7-32.0) | 0.121 |
| Etiology (n, %)   - ALD - Viral - Mix (ALD + viral) - NASH - CHOL - OTHER | 22 (44.9)  9 (18.4)  6 (12.2)  2 (4.1)  2 (4.1)  8 (16.3) | 59 (43.4)  27 (19.9)  10 (7.3)  11 (8.1)  6 (4.4)  23 (16.9) | 15 (30.6)  11 (22.4)  2 (4.1)  12 (24.5)  1 (2.1)  8 (16.3) | 0.094 |
| Decompensation (n, %) | 41 (83.7) | 77 (56.6) | 20 (40.8) | **<0.001** |
| HVPG (mmHg) | 19 (16-23) | 18 (13-20) | 12 (9-18) | **<0.001** |
| Varices (n, %)   - None - Small - Large - (Unknown) | 17 (34.7)  13 (26.5)  19 (38.8)  0 (0) | 42 (30.9)  37 (27.2)  55 (40.4)  2 (1.5) | 20 (40.8)  10 (20.4)  14 (28.6)  5 (10.2) | 0.571 |
| Child score (points) | 8 (8-10) | 6 (5-7) | 5 (5-6) | **<0.001** |
| MELD (points) | 16 (13-18) | 10 (9-12) | 9 (7-12) | **<0.001** |
| VCTE (kPa)^1^ | 49.7 (25.0-75.0) | 32.7 (18.8-61.7) | 20.4 (14.1-33.1) | **<0.001** |
| VitD (nmol/L) | 31.7 (19.9-47.3) | 44.2 (23.6-68.8) | 56.1 (32.9-76.2) | **<0.001** |
| VitE (µmol/L) | 21.9 (17.8-26.5) | 23.2 (19.3-28.5) | 25.7 (21.9-30.0) | **0.003** |
| PLT (G/L) | 81 (58-120) | 101 (70-136) | 133 (98-164) | **<0.001** |
| Albumin (g/L) | 30.2 (26.6-33.8) | 37.0 (34.3-40.2) | 40.1 (38.5-42.8) | **<0.001** |
| Bilirubin (mg/dL) | 2.29 (1.50-3.22) | 1.03 (0.66-1.51) | 0.80 (0.63-1.02) | **<0.001** |
| INR | 1.7 (1.5-1.9) | 1.3 (1.2-1.5) | 1.2 (1.1-1.3) | **<0.001** |
| AT-III activity (%)^2^ | 43 (36-50) | 68 (57-77) | 83 (70-94) | **<0.001** |
| Protein-C activity (%)^3^ | 33 (27-42) | 59 (46-72) | 84 (73-104) | **<0.001** |
| BA (µmol/L)^4^ | 41.7 (26.3-73.3) | 14.3 (8.4-32.5) | 7.5 (4.6-14.2) | **<0.001** |
| ELF^5^ | 12.5 (11.9-13.1) | 11.2 (10.5-12.1) | 10.5 (9.8-11.2) | **<0.001** |

^1^ Reliable VCTE results were available in N=174 (74.4%) patients: n=31 in Q1, n=100 in Q2-Q4, n=37 in Q5.
^2^ AT-III activity was available in N=230 (98.3%) patients.
^3^ Protein C activity was available in N=229 (97.9%) patients.
^4^ Bile acid serum levels were available in N=224 (95.7%) patients.
^5^ ELF score was available in N=219 (93.6%) patients.

P-values <0.05 are indicated in bold. Abbreviations: (M) male sex, (ALD) alcohol-related liver disease, (NASH) non-alcoholic steatohepatitis, (CHOL) cholestatic liver disease, (HVPG) hepatic venous pressure gradient, (VCTE) vibration-controlled transient elastography, (BMI) body-mass index, (MELD) Model for end-stage liver disease, (PLT) platelet count, (INR) international normalized ratio, (TPZ) thromboplastin time, (vWF) von Willebrand factor, (AT-III) antithrombin III, (BA) bile acids, (ELF) enhanced liver fibrosis score

**Supplementary table-S4. Independent risk factors for moderate/severe vitamin A deficiency in two different models using Child-Turcotte-Pugh score (Model 1) or hepatic venous pressure gradient (Model 2).**

| **Parameter** | **Univariate analysis** | | | **Multivariate analysis** | | |
| --- | --- | --- | --- | --- | --- | --- |
| **Model 1 (CTP)** | **OR** | **95% CI** | **P-value** | **OR** | **95% CI** | **P-value** |
| Sex (male) | 0.63 | 0.36-1.08 | 0.092 | 0.78 | 0.38-1.62 | 0.511 |
| Age (per year) | 0.97 | 0.94-0.99 | **0.003** | 0.95 | 0.92-0.98 | **0.001** |
| CTP score (per point) | 2.44 | 1.89-3.15 | **<0.001** | 2.40 | 1.80-3.22 | **<0.001** |
| Elevated BA levels  (>10µmol/L) | 7.06 | 3.73-13.4 | **<0.001** | 4.22 | 2.00-8.90 | **<0.001** |
| **Parameter** | **Univariate analysis** | | | **Multivariate analysis** | | |
| **Model 2 (HVPG)** | **OR** | **95% CI** | **P-value** | **OR** | **95% CI** | **P-value** |
| Sex (male) | 0.63 | 0.36-1.08 | 0.092 | 0.75 | 0.39-1.42 | 0.374 |
| Age (per year) | 0.97 | 0.94-0.99 | **0.003** | 0.96 | 0.93-0.98 | **0.002** |
| HVPG (mmHg) | 1.16 | 1.10-1.22 | **<0.001** | 1.12 | 1.06-1.19 | **<0.001** |
| Elevated BA levels  (> 10µmol/L) | 7.06 | 3.73-13.4 | **<0.001** | 4.46 | 2.20-9.05 | **<0.001** |

P-values <0.05 are indicated in bold. Abbreviations: (OR) odds ratio, (95%CI) 95% confidence interval, (HVPG) hepatic venous pressure gradient, (CTP) Child-Turcotte-Pugh, (BA) bile acids

**Supplementary table-S5. Independent risk factors for vitamin D deficiency in two different models using Child-Turcotte-Pugh score (Model 1) or hepatic venous pressure gradient (Model 2).**

| **Parameter** | **Univariate analysis** | | | **Multivariate analysis** | | |
| --- | --- | --- | --- | --- | --- | --- |
| **Model 1 (CTP)** | **OR** | **95% CI** | **P-value** | **OR** | **95% CI** | **P-value** |
| Sex (male) | 1.26 | 0.73-2.17 | 0.400 | - | - | - |
| Age (per year) | 0.98 | 0.96-1.01 | 0.175 | - | - | - |
| CTP score (per point) | 1.40 | 1.17-1.67 | **<0.001** | 1.38 | 1.14-1.66 | **<0.001** |
| Elevated BA levels  (> 10µmol/L) | 1.62 | 0.93-2.83 | 0.089 | 1.11 | 0.61-2.03 | 0.733 |
| **Parameter** | **Univariate analysis** | | | **Multivariate analysis** | | |
| **Model 2 (HVPG)** | **OR** | **95% CI** | **P-value** | **OR** | **95% CI** | **P-value** |
| Sex (male) | 1.26 | 0.73-2.17 | 0.400 | - | - | - |
| Age (per year) | 0.98 | 0.96-1.01 | 0.175 | - | - | - |
| HVPG (mmHg) | 1.06 | 1.02-1.11 | **0.008** | 1.11 | 1.05-1.18 | **<0.001** |
| Elevated BA levels  (> 10µmol/L) | 1.62 | 0.93-2.83 | 0.089 | 1.17 | 0.63-2.17 | 0.629 |

P-values <0.05 are indicated in bold. Abbreviations: (OR) odds ratio, (95%CI) 95% confidence interval, (HVPG) hepatic venous pressure gradient, (CTP) Child-Turcotte-Pugh, (BA) bile acids

**Supplementary table-S6. Independent risk factors for vitamin E deficiency.**

| **Parameter** | **Univariate analysis** | | | **Multivariate analysis** | | |
| --- | --- | --- | --- | --- | --- | --- |
|  | **OR** | **95% CI** | **P-value** | **OR** | **95% CI** | **P-value** |
| Sex (male) | 1.33 | 0.25-7.04 | 0.734 | - | - | - |
| Age (per year) | 0.95 | 0.90-1.01 | 0.105 | - | - | - |
| HVPG (mmHg) | 1.03 | 0.91-1.16 | 0.648 | - | - | - |
| CTP score (per point) | 1.23 | 0.85-1.78 | 0.281 | - | - | - |
| Elevated BA levels  (> 10µmol/L) | 1.32 | 0.25-6.97 | 0.743 | - | - | - |

Abbreviations: (OR) odds ratio, (95%CI) 95% confidence interval, (HVPG) hepatic venous pressure gradient, (CTP) Child-Turcotte-Pugh, (BA) bile acids

**Supplementary table-S7. Factors independently associated with decompensated advanced chronic liver disease (dACLD).**

| **Parameter** | **Univariate analysis** | | | **Multivariate analysis** | | |
| --- | --- | --- | --- | --- | --- | --- |
|  | **OR** | **95% CI** | **P-value** | **OR** | **95% CI** | **P-value** |
| Sex (male) | 1.06 | 0.62-1.83 | 0.830 | - | - | - |
| Age (years) | 1.01 | 0.99-1.03 | 0.357 | - | - | - |
| HVPG (mmHg) | 1.25 | 1.17-1.33 | **<0.001** | 1.19 | 1.11-1.27 | **<0.001** |
| MELD (per point) | 1.37 | 1.24-1.52 | **<0.001** | 1.22 | 1.09-1.36 | **<0.001** |
| Vitamin A (µmol/L) | 0.15 | 0.08-0.31 | **<0.001** | 0.50 | 0.22-1.13 | 0.094 |
| Vitamin D (nmol/L) | 0.99 | 0.98-1.00 | **0.031** | 1.00 | 0.99-1.01 | 0.705 |

Abbreviations: (OR) odds ratio, (95% CI) 95% confidence interval, (HVPG) hepatic venous pressure gradient, (MELD) Model for end-stage liver disease, (CSPH) clinically significant portal hypertension

**SUPPLEMENTARY FIGURES**

**Supplementary figure-S1. Patient flow chart**


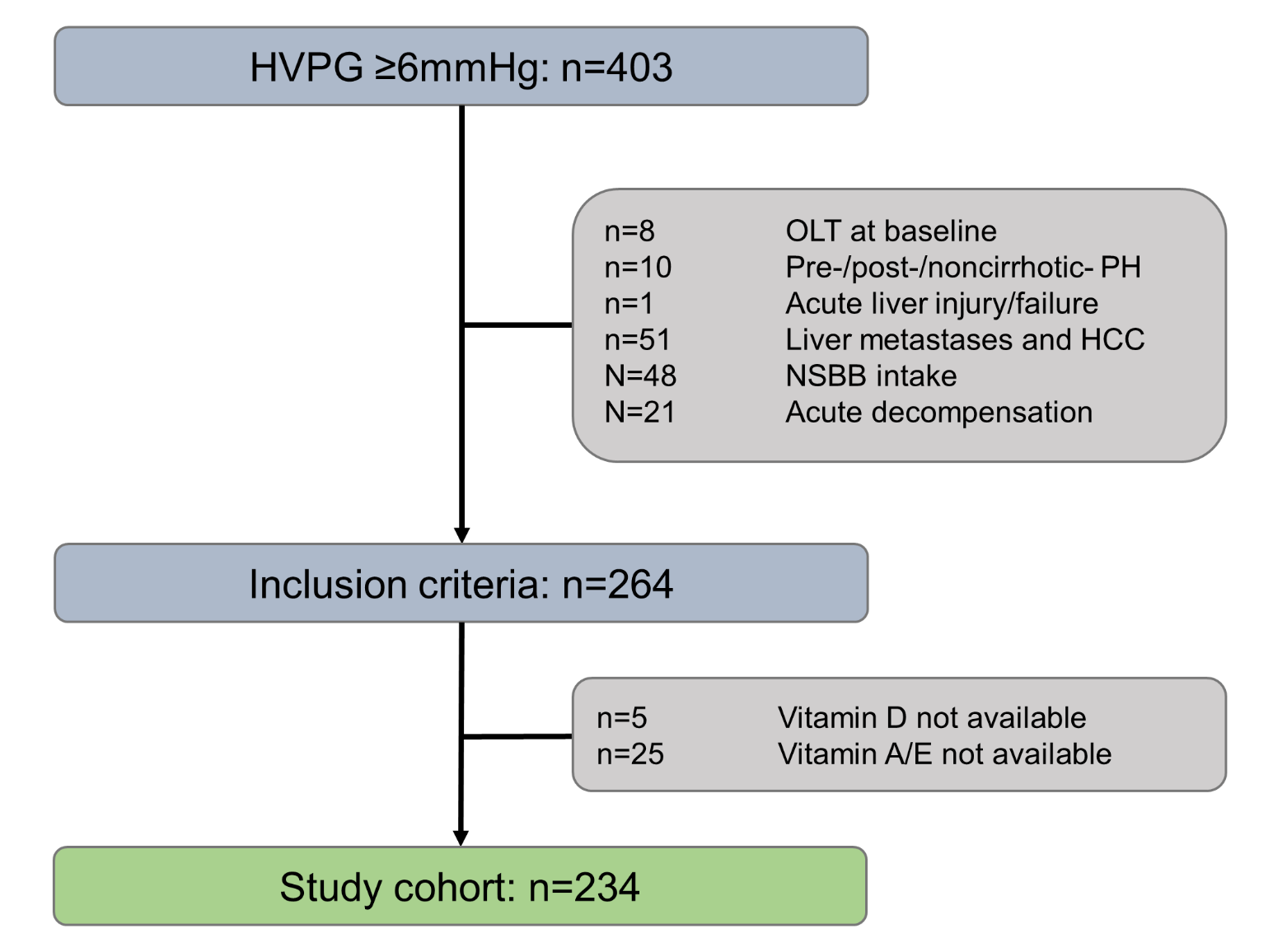


Abbreviations: (HVPG) hepatic venous pressure gradient; (OLT) orthotopic liver transplantation; (PHT) portal hypertension; (NCPH) non-cirrhotic portal hypertension; (NSBB) non-selective betablockers

**Supplementary figure-S2. Vitamin D serum levels in patients stratified by (A) Child-Turcotte-Pugh (CTP) stage and (B) hepatic venous pressure gradient (HVPG), and the reported intake of prescription vitamin D supplements.**


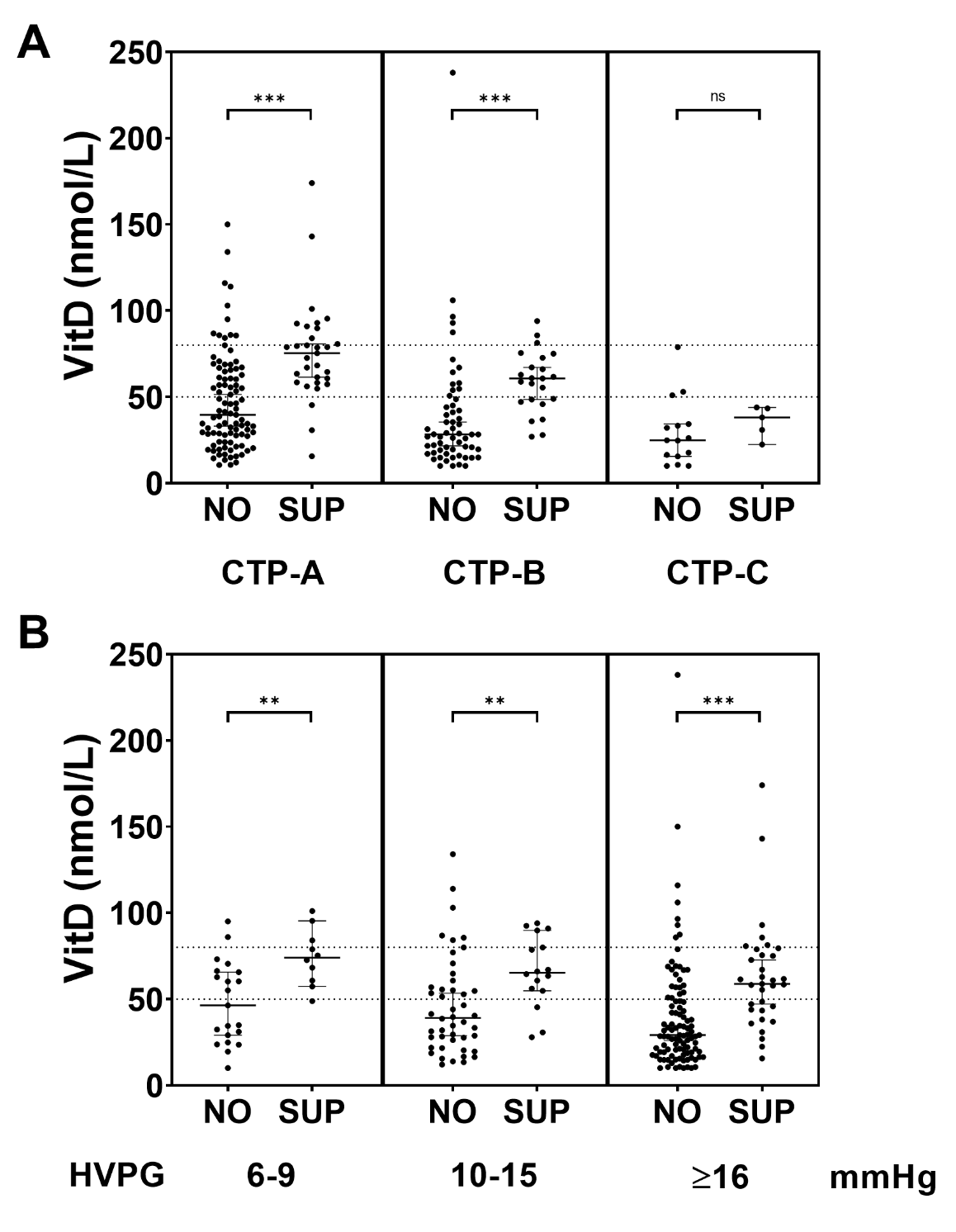


Legend: (ns) not significant; (*) P<0.05; (**) P<0.01; (***) P<0.001. Dotted lines indicate cut-offs for vitamin D insufficiency and deficiency, respectively. Patients with insufficient data on prescription vitamin supplements (n=6) were excluded for this analysis. Abbreviations: (VitD) vitamin D; (CTP) Child-Turcotte-Pugh; (HVPG) hepatic venous pressure gradient; (NO) no VitD supplementation; (SUP) prescription of VitD supplementation

**Supplementary figure-S3. Correlation of Child-Turcotte-Pugh score and coagulation laboratory parameters with vitamin A serum levels.**


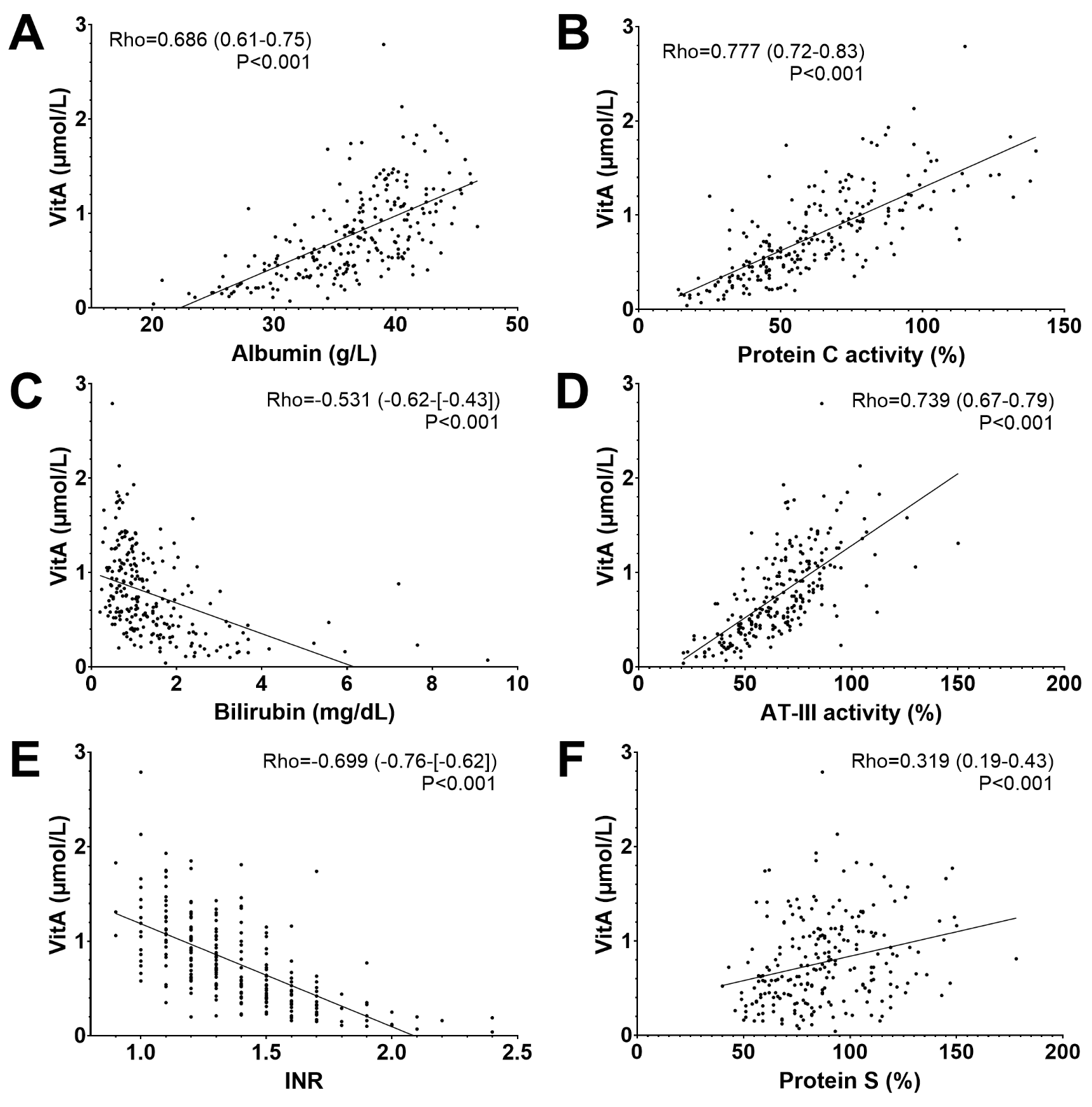


Abbreviations: (VitA) vitamin A; (INR) international normalized ratio; (AT-III) antithrombin-III

**SUPPLEMENTARY REFERENCES**

1. Reiberger, T., et al., *Measurement of the Hepatic Venous Pressure Gradient and Transjugular Liver Biopsy.* Journal of Visualized Experiments, 2020(e58819. In-press (2020).).

2. Schwabl, P., et al., *New reliability criteria for transient elastography increase the number of accurate measurements for screening of cirrhosis and portal hypertension.* Liver Int, 2015. **35**(2): p. 381-90.

3. Organization), W.W.H., *Serum retinol concentrations for determining the prevalence of vitamin A deficiency in populations. .* 2011(Vitamin and Mineral Nutrition Information System. ).

4. Holick, M.F., *Vitamin D deficiency.* N Engl J Med, 2007. **357**(3): p. 266-81.

5. Simbrunner, B., et al., *Non-invasive detection of portal hypertension by enhanced liver fibrosis score in patients with different etiologies of advanced chronic liver disease.* Liver Int, 2020.
